# Expression of MET in circulating tumor cells correlates with expression in tumor tissue from advanced-stage lung cancer patients

**DOI:** 10.18632/oncotarget.15345

**Published:** 2017-02-15

**Authors:** Marius Ilie, Edith Szafer-Glusman, Véronique Hofman, Elodie Long-Mira, Rebecca Suttmann, Walter Darbonne, Catherine Butori, Salomé Lalvée, Julien Fayada, Eric Selva, Wei Yu, Charles-Hugo Marquette, David S. Shames, Elizabeth Punnoose, Paul Hofman

**Affiliations:** ^1^ Laboratory of Clinical and Experimental Pathology and Liquid Biopsy Laboratory, Pasteur Hospital, University Hospital Federation OncoAge, Université Côte d'Azur, Nice, France; ^2^ Institute for Research on Cancer and Ageing, Nice (IRCAN), INSERM U1081/UMR CNRS 7284, Team 3, Antoine Lacassagne Cancer Center, Nice, France; ^3^ Department of Oncology Biomarker Development and Oncology Clinical Development, Genentech, Inc, South San Francisco, California, USA; ^4^ Nice Hospital-Related Biobank (BB 0025-00033), Pasteur Hospital, Nice, France; ^5^ Pneumology Department, Pasteur Hospital, Nice, France

**Keywords:** MET protein expression, circulating tumor cells, NSCLC, immunocytochemistry, targeted therapy

## Abstract

Given the difficulty in obtaining adequate tissue in NSCLC, we investigated the utility of circulating tumor cells (CTCs) for MET status assessment in NSCLC patients. We used two platforms for CTC capture, and assessed MET expression in CTCs and matched-bronchial biopsies in patients with advanced-stage III/IV lung adenocarcinoma. Baseline peripheral blood was collected from 256 advanced-stage III/IV NSCLC patients from Genentech clinical trials, and from 106 patients with advanced-stage III/IV lung adenocarcinoma treated at the Department of Pneumology, Pasteur Hospital, Nice. CTCs were enriched using CellSearch (Genentech), or ISET technologies (Pasteur Hospital). MET expression was evaluated by immunofluorescence on CellSearch, and by immunocytochemistry on ISET-enriched CTCs and on matched FFPE tissue sections (Pasteur Hospital). CTCs were detected in 83 of 256 (32%) patients evaluated on CellSearch, with 30 samples (12%) exhibiting ≥ 5 CTCs/7.5 ml blood. On ISET, CTC were observed in 80 of 106 patients (75%), and 79 patients (75%) exhibited ≥ 5 CTCs/4 ml blood. MET expression on ISET CTCs was positive in 72% of cases, and the MET expression on matched-patient tissue was positive in 65% patients using the Onartuzumab IHC scoring algorithm (93% concordance). Quantification of MET expression using H-scores showed strong correlation between MET expression in tissue and CTCs (Spearman correlation, 0.93). MET status in CTCs isolated on ISET filters from blood samples of advanced-stage NSCLC patients correlated strongly with MET status in tumor tissue, illustrating the potential for using CTCs as a non-invasive, real-time biopsy to determine MET status of patients entering clinical trials.

## INTRODUCTION

Non-small cell lung cancer (NSCLC) accounts for approximately 80% of lung cancers and remains a major cause of cancer death worldwide [1]. In the last few decades, better understanding of the molecular pathophysiology of NSCLC has led to the development of selective agents to specifically target key oncogenic drivers with improved outcome rates [2]. Genomic-targeted therapy to *EGFR* gene mutations (TKI), *EML4–ALK* fusion and *ROS1* translocations, have proven marked treatment responses being more effective than conventional chemotherapies in advanced NSCLC patients [3, 4]. However, despite the benefits from EGFR-TKIs, almost all patients will ultimately develop resistance, with dysregulation of MET observed in up to 20% of resistance cases [5]. Dysregulation of the MET signaling pathway has been reported in several types of cancer, in particular in NSCLC, and is associated with tumor growth, survival, motility and migration, epithelial to mesenchymal transition, and, ultimately, invasion [6–8]. The MET abnormalities include MET protein overexpression, gene amplification or mutation [9]. Overexpression of MET protein in tumor tissue relative to adjacent normal tissues occurs in 25 to 75% of NSCLC, however, its association with patients’ outcome remains controversial [10–14]. Several clinical trials have demonstrated that MET protein overexpression could be used as a biomarker for acquired resistance to EGFR-TKIs, and an association of MET and EGFR dual inhibitory strategies showed a synergistic benefit in MET protein overexpression patients with acquired resistance to EGFR-TKIs [15].

MET receptor tyrosine kinase has emerged as a potential therapeutically relevant target in NSCLC [16, 17]. A number of MET tyrosine-kinase inhibitors are currently undergoing testing in early-phase clinical trials [17, 18]. A new MET-targeting inhibitor, INC280, has shown promising results in a phase I clinical trial reported at the 2016 American Society of Clinical Oncology meeting [19]. In this study, preliminary efficacy was seen in NSCLC patients with high MET expression and wild-type EGFR [19]. In addition, although crizotinib, initially designed as a MET inhibitor, is FDA-approved for *ALK*-rearranged NSCLC patients, it has shown activity in a subgroup of NSCLC patients that lack an ALK translocation but overexpress MET, or carrying *de novo* MET gene amplification [20–22].

Eligibility of patients to targeted therapies relies on diagnostic assays performed on a tumor biopsy. This invasive procedure is associated with a relative high risk of morbidity, and inoperable patients can be deprived from potentially more efficient therapies [23]. Moreover, for most advanced NSCLC patients, testing is often limited by insufficient tissue, thus, there is a need of alternative, noninvasive methods for diagnostic assessment [23].

Circulating tumor cells (CTCs) represent an accessible, non-invasive surrogate tissue that allows access to biomarker assessment in vulnerable lung cancer patients for whom tissue biopsies are inaccessible or extremely difficult to perform and to repeat [23]. Among the commercially available CTC platforms, CellSearch captures CTCs expressing EpCAM [23], while ISET captures CTCs based on cell size (filtration) [23]. Selection of a CTC capture methodology should take into account the sensitivity of the isolation technology, the specificity in the diagnosis of circulating cells with malignant features, and the suitability for downstream molecular analyses.

To investigate the utility of a liquid biopsy to assess a patient's lung tumor's MET status, here we evaluated the prevalence of MET expression in CTCs using 2 different CTC platforms, CellSearch and ISET, and compared MET expression in CTCs and matched tumor tissue in a retrospective cohort of 80 advanced-stage NSCLC patients.

## RESULTS

### CTC counts and poor MET expression in CTCs detected by CellSearch

**Figure 1:**
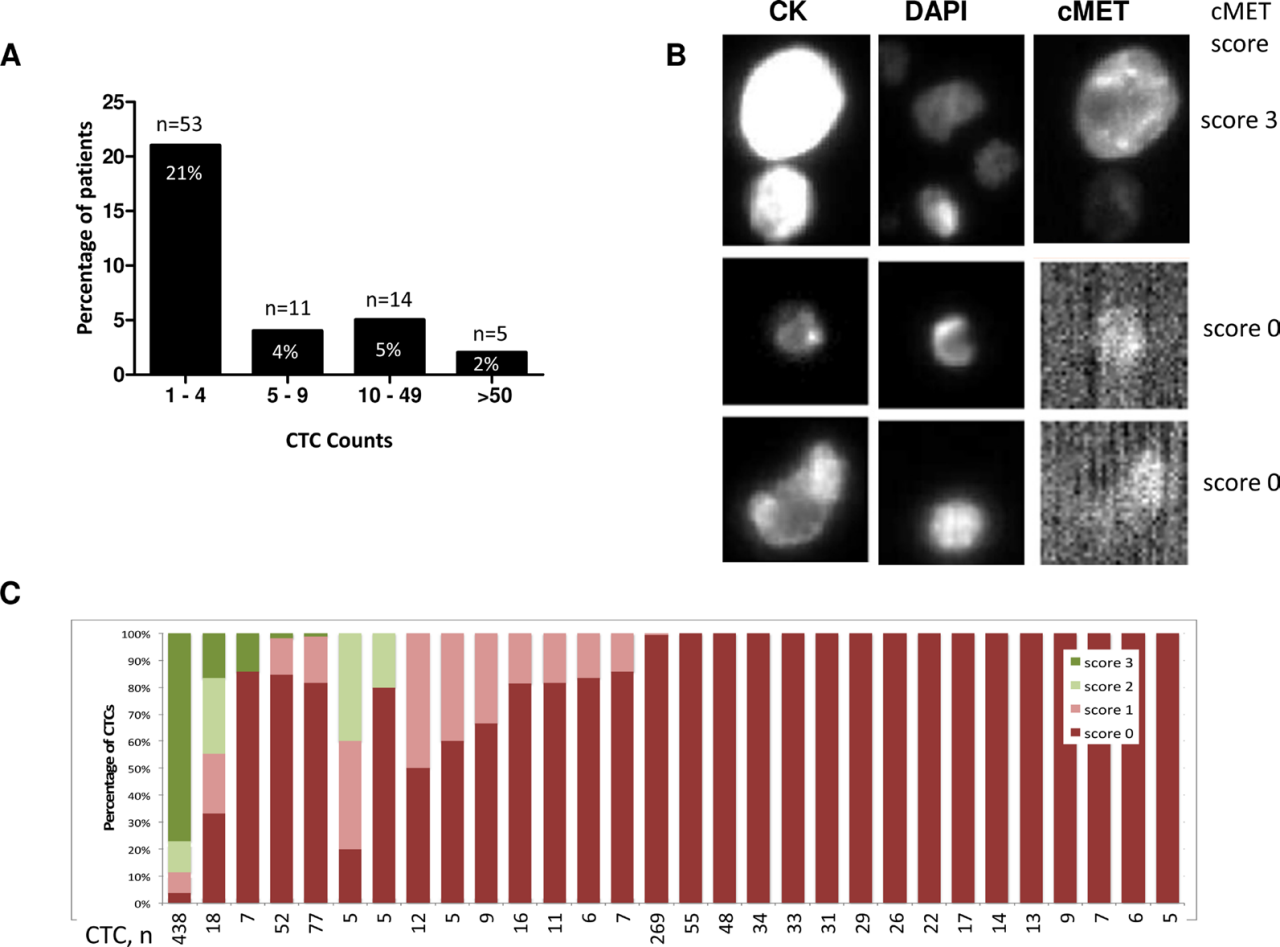
CTC enumeration and MET protein staining in blood samples evaluated on the CellSearch system (**A**) Distribution of CTC counts per 7.5 ml blood in 256 patients, indicating the number and percentage of patients in each CTC enumeration subgroup (173 patients with CTCs = 0 are not shown). (**B**) Representative examples of MET protein staining in CTCs. Upper panel, CTC exhibiting strong MET expression (score 3+), medium and bottom panels, no MET staining detected (score 0). (**C**) Distribution of MET scores in CTCs from patients with ≥ 5 CTCs/7.5 ml, *n* = total number of CTCs detected in each sample.

Baseline blood samples from 256 Stage III/IV NSCLC patients were evaluated for CTC enumeration on the CellSearch platform. CTC enumeration ranged from 0 to > 200 CTCs in 7.5 ml blood. One or more CTCs were observed in 83 patients (32%), with 30 samples (12%) exhibiting ≥ 5 CTCs/7.5 ml blood (Figure 1A, Table 1). The level of MET protein expression in CTCs was evaluated in all samples that had at least 1 detected CTC. MET protein expression was low in CTCs, with most cells scoring 0, and only 9 patients having CTCs with medium to high MET expression (scores 2+ or 3+), (Table 1, representative examples in Figure 1B). H-scores ranged from 0 to 262, with 90% of samples exhibiting H-scores below 100, consistent with low MET expression. From the 30 patients with ≥ 5 CTCs, 7 samples had CTCs scoring 2+ or 3+, and in only 1 patient these represented more than 50% of the CTC population (Figure 1C). The low CTC counts and poor MET detection in CTCs tested on the CellSearch platform prompted us to evaluate alternative approaches for CTC enumeration and MET assessment in NSCLC patients.

**Table 1:** Clinical and pathological characteristics of 256 patients from NCT01519804 and NCT01496742 trials included in our study

Patients demographics	NCT01519804 (*n* = 72*)	NCT01496742 (*n* = 184*)	All patients (*n* = 256*)
**Age**			
Median (range)	66.5 (46–84)	63 (19–82)	64 (19–84)
**Sex**			
Male	54 (75%)	99 (53.8%)	153 (59.8%)
Female	18 (25%)	85 (46.2%)	103 (40.2%)
**Tobacco Use History**			
Former or Current smoker	70 (97.2%)	147 (79.9%)	217 (84.8%)
Never smoked	2 (2.8%)	37 (20.1%)	39 (15.2%)
**Histology**			
Squamous NSCLC	72 (100%)		72 (28.1%)
Non-squamous NSCLC		184 (100%)	184 (71.9%)
**ECOG Performance Status**	*n* = 72	*n* = 181	*n* = 253
0	29 (40.3%)	92 (50.8%)	121 (47.8%)
1	43 (59.7%)	89 (49.2%)	132 (52.2%)
**CTC enumeration**			
≥ 5 CTCs	*n* = 9 (12.5%)	*n* = 21 (11%)	*n* = 30
Met scores 2+, 3+	*n* = 3 (4%)	*n* = 6 (3%)	*n* = 9

* only patients evaluated for CTC were included in the table.

### CTCs detected by blood sample enumeration on the ISET platform

**Figure 2:**
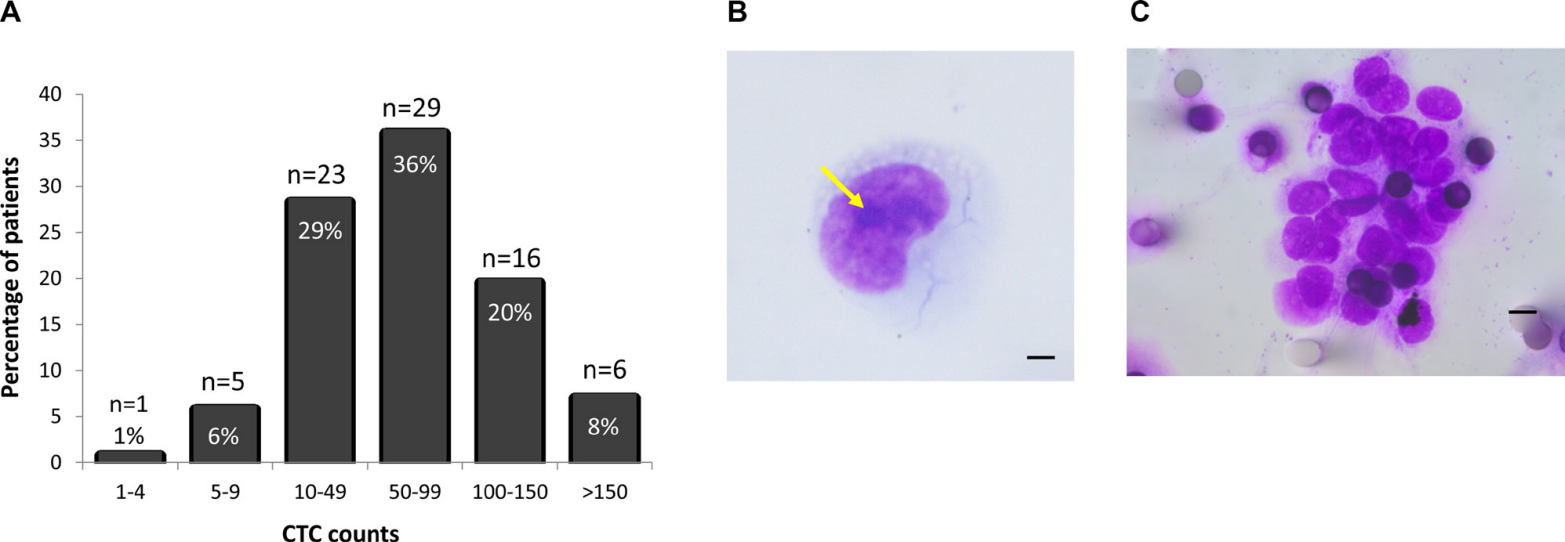
(**A**) Distribution of CTC counts in 4 ml blood from patients with advanced-stage NSCLC filtered on the ISET system. (**B**) Example of an isolated CTC with malignant cytomorphological features (Arrow, nucleoli; Original magnification ×1000; bar, 10 μm). (**C**) Circulating tumor cluster composed of more than 20 CTCs with malignant cytomorphological features (Original magnification ×1000; bar, 8 μm).

Peripheral blood samples were collected from 106 patients with advanced stage III/IV lung adenocarcinoma. 99 patients (93%) were chemotherapy naïve, and 7 (7%) had neoadjuvant chemotherapy. Blood samples were processed within one hour of collection using the ISET filtration system. Filters were either stored at −20^°^C for up to 180 days, or immediately stained with MGG on 4 out of the 10 filter-spots (see Methods). These 4 spots, enriched in the filtrate from 4 ml blood, were examined for the presence of CNHC. CTC enumeration was computed as the number of CNHC-MF and CNHC-UMF cells identified in 4 ml blood. CNHC-MF and/or CNHC-UMF were detected in 80 of 106 (75%) patient samples examined. 79 of the 80 CTC-positive samples exhibited more than 5 CTCs per 4 ml blood (Figure 2A), with a median of 60 CTCs and a max. of 256 CTCs per 4 ml blood.

A representative example of a circulating cell with malignant features, characterized by a large size (diameter larger than 20 μm), large nuclei and presence of nucleoli is shown in Figure 2B. Clusters of CTCs (also known as circulating tumor microemboli), defined as aggregates containing two or more tightly juxtaposed malignant cells (median 5 cells/cluster) (Figure 2C), were observed in 75 of the 80 CTC-positive patients (94%), with a number of clusters ranging from 1 to 23 per 4 ml sample, and a median of 5 clusters per 4 ml blood sample.

### MET expression on CTCs enriched on ISET filters

**Figure 3:**
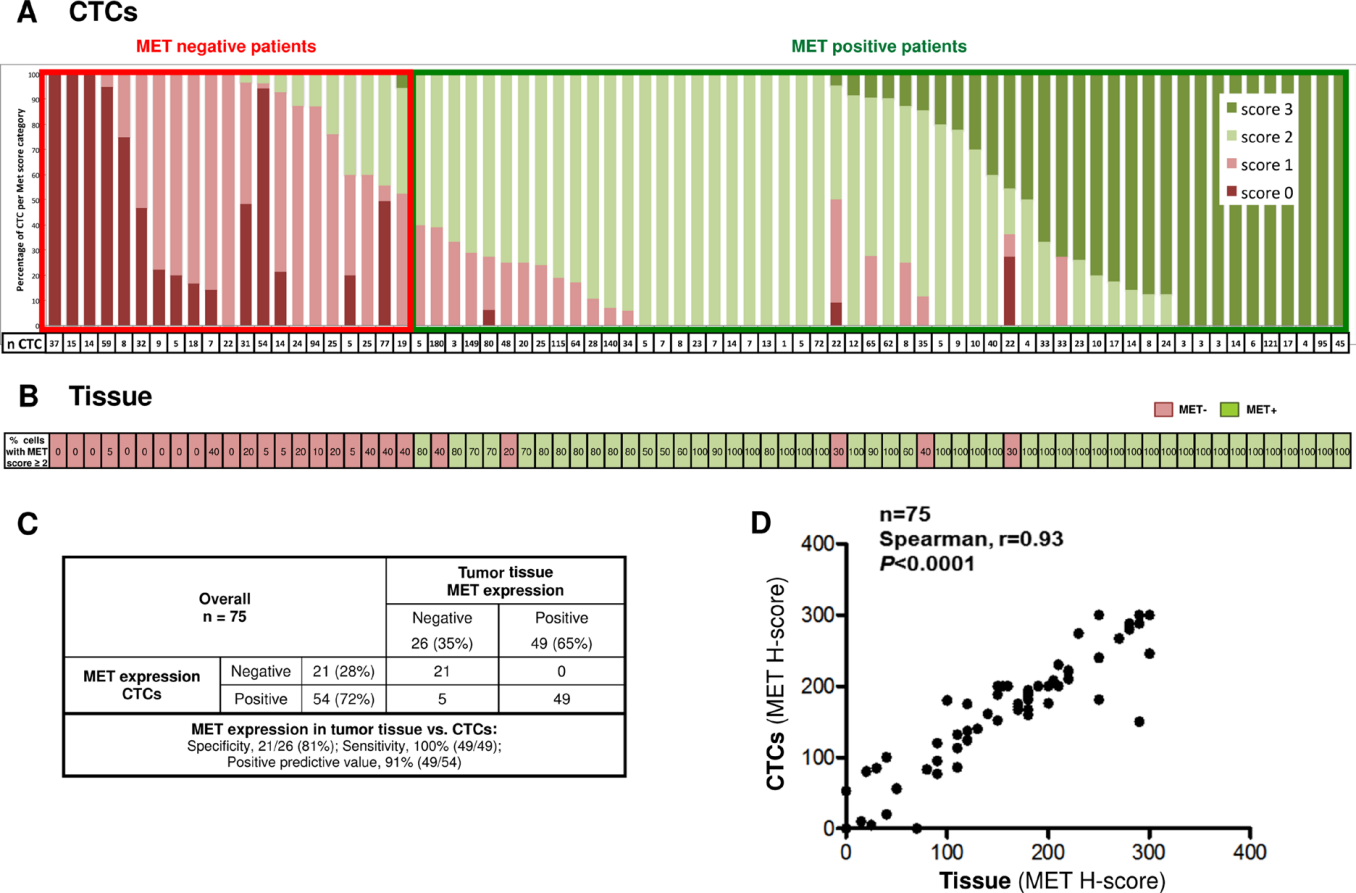
(**A**) Distribution of MET scores in CTCs from NSCLC patients analyzed on the ISET system. (**B**) MET expression in FFPE tissue slides for the same patients. Numbers indicate the percentage of tumor cells with MET protein intensity ≥ 2+ (2+ or 3+). Red, MET negative tumors (≥ 50% tumor with MET intensity 0+ or 1+); Green, MET positive tumors (≥ 50% tumor with MET intensity 2+ or 3+). (**C**) Concordance of MET expression in tumor tissue and corresponding CTCs from 75 NSCLC patients. (**D**) Linear concordance between MET H-scores in CTCs *vs*. tumor tissue.

**Figure 4:**
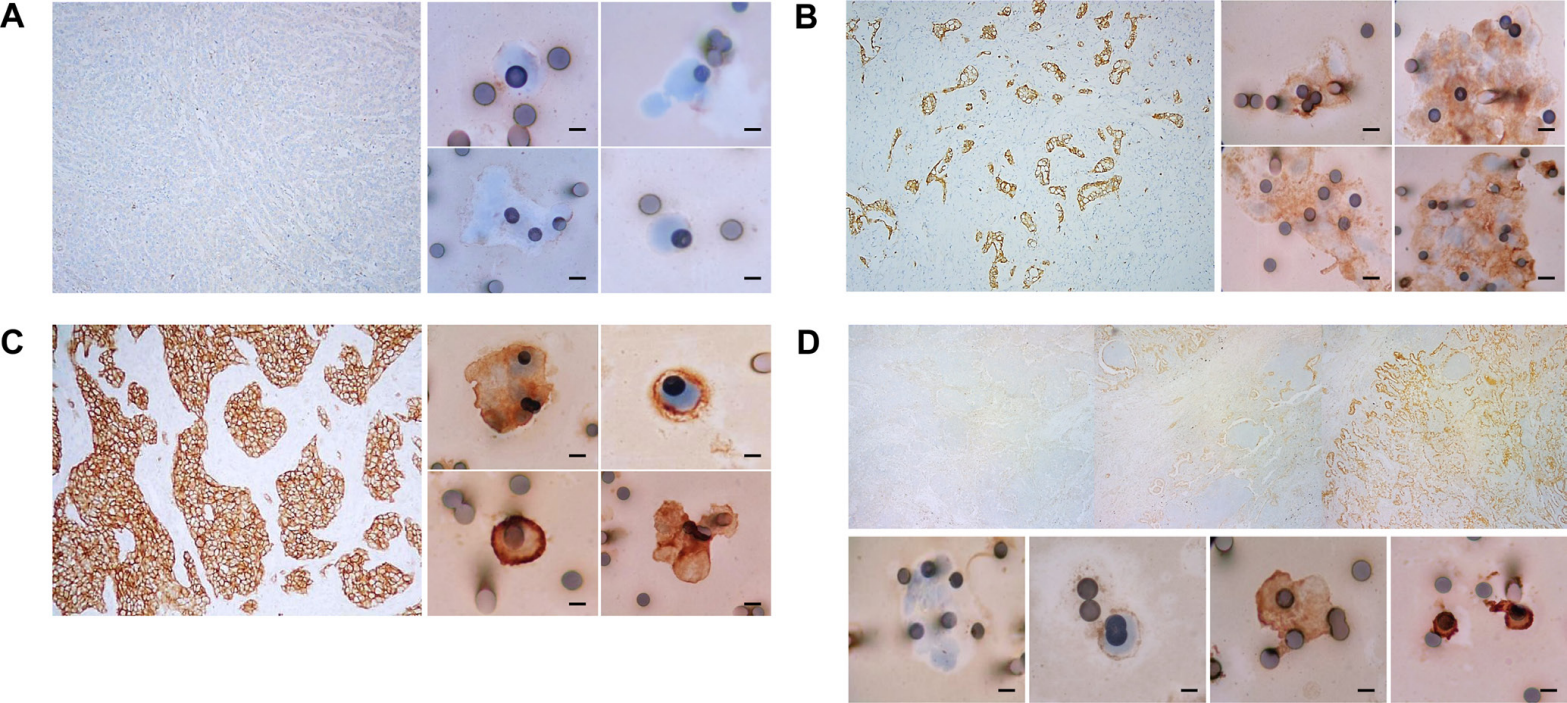
MET protein staining in tumor tissue and corresponding CTCs from selected NSCLC patients (**A**) Patient with negative MET staining in tumor tissue (*left panel*, Original magnification ×100), and negative MET staining in CTCs (*right panel*, Original magnification ×400; bar, 8 μm). (**B**) Patient with intermediate (2+) MET staining in tumor tissue (*left panel*, Original magnification ×100), and in CTCs (*right panel*, Original magnification ×400; bar, 8 μm). (**C**) Patient with strong (3+) MET staining in tumor tissue (*left panel*, Original magnification ×100), and concordant high MET staining in CTCs (*right panel*, Original magnification ×400; bar, 8 μm). (**D**) Patient exhibiting discordant MET staining in tumor tissue (H-score, 100; *upper panel*, representative IHC gallery optically scanned at ×100) and corresponding CTCs (H-score, 180; *lower panel*, 4-picture gallery with CTCs stained 0, 1+, 2+ or 3+; Original magnification ×400; bar, 8 μm).

The ICC assay to evaluate MET expression on CTCs was developed using a panel of human cell lines that express low, medium and high levels of MET. Cell-lines spiked into healthy donors’ blood were enriched on ISET filers and stained for MET. Staining conditions were optimized to result in concordant MET scores for cell-blocks (IHC) and the corresponding blood spike-ins (ICC). The progressive increase in MET staining intensities from low- to high-MET expressing cells observed in cell blocks was paralleled by a progressive increase in CTC staining intensity in the corresponding spike-in samples (Supplementary Figure 2). Next, we used the optimized method to evaluate MET expression in three unstained spots from each of the 80 ISET enriched patient samples selected. Examination of the MET stained filter identified 75 samples with at least one cell with characteristics of CTCs in the 3-filter spots, with a median of 19 CTCs per 3 ml blood (range: 1–180). For each patient, MET was scored in all the CTCs that were detected on the 3 stained spots. MET scores in CTCs were often homogeneous within a patient, with a mixture of CTCs scoring on adjacent intensity levels (e.g. 0 and 1, 1 and 2, 2 and 3, Figure 3A). The intensity of MET staining was similar for CTC-clusters and single cells from the same sample (Figure 4). A representative example of MET staining in a large cluster is observed in Figure 4B, and examples of smaller clusters are observed in Figure 4C and 4D. Using the Onartuzumab scoring algorithm, 54 of the 75 (72%) patients’ blood samples were defined as MET positive (Figure 3A).

### MET expression in CTCs correlated strongly with MET status in patient-matched tissue

MET IHC was evaluated in FFPE tissue samples from patient-matched biopsies. Applying the Onartuzumab algorithm, MET patient status was positive in 49 of 75 (65%) patients’ tissue samples (Figure 3B). Comparison of MET protein status in CTCs and tissue revealed concordant positive or negative MET classification in 70 of the 75 (93%) patients evaluated (Figure 3C). Representative examples of concordant MET expression in CTCs and tumor tissue are shown in Figure 4A (MET Negative in CTCs and tissue), Figure 4B and 4C (MET positive in CTC and tissue). From 54 patients classified as MET positive in CTCs, 49 showed MET positive tissue (Positive predictive value = 91%, Figure 3C). All 49 patients that were positive in tissue were also positive in CTCs (Sensitivity =100%), and only 5 patients were discordant, with MET status positive in CTCs, and negative in tissue (Figure 3C, representative example in Figure 4D).

To get a more finely graded picture of MET expression in the tumor tissue and CTCs, we also computed MET expression using an H-scoring system that takes into consideration the staining intensity and the proportion of CTCs exhibiting each MET score. Comparison of patient-matched tumor-tissue and blood showed a linear correlation between MET H-scores in tumor-biopsies and CTCs (Figure 3D), with a median H score of 180 in Tumor tissue and 189 in CTCs.

### Correlation of CTC enumeration and MET expression with clinico-pathological variables and clinical outcome

CTC enumeration was a prognostic factor in the Pasteur cohort: using a cutoff of 50 CTCs [24], we found a significant correlation between CTC enumeration and worse PFS (Supplementary Figure 3A). There was no relationship between MET expression by IHC and clinico-pathological variables of patients (age, *P*-value = 0.159; gender, *P*-value = 0.088; smoking status, *P*-value = 0.280; tumor size, *P*-value = 0.089). At a median follow up of 29 months, 46 patients had progressed and 26 patients had died. MET expression in tumor tissue or CTCs did not correlate with PFS in univariate survival analysis (Supplementary Figure 3B).

In NSCLC patients evaluated for CTCs on CellSearch, a prognostic value of CTC counts was reported using a cutoff of 5 CTCs [25]. The small number of patients with ≥5 CTC in the separate treatment arms from NCT01519804 (*n* = 6 CTC positive patients in placebo arm) and NCT01496742 studies (*n* = 2 and 7 CTC positive patients in the placebo arms of the 2 regimens evaluated), and the even smaller number of patients with MET expression in CTCs precludes any robust analysis of the value of CTCs in these studies.

## DISCUSSION

In this study we evaluated the potential utility of CTCs for assessment of MET in advanced NSCLC patients. We tested two approaches for CTC analysis that differed in the platform for CTC enrichment (CellSearch *vs*. ISET) and MET evaluation methodology (immunofluorescence *vs*. immunohistochemistry), and also in blood stabilizing agents and time from blood collection to processing. In the CellSearch-based approach used for Genentech trials’ samples, blood was collected in CellSave tubes and shipped to a central lab for CTC enumeration, a process that lasted from 18 to more than 96 hours. In the ISET-based approach used for the Pasteur Hospital samples, blood was collected in EDTA tubes and processed for CTC enumeration within 1 hour of collection. We found that the ISET approach identified a higher proportion of CTC-positive patients than the CellSearch approach. This was consistent with previous reports demonstrating high sensitivity for CTC enrichment using the ISET methodology in blood samples from NSCLC patients [24, 25]. Because of the different overall approaches utilized, it is not possible to unequivocally identify which step of the whole processes contributed most to the higher success of CTC enumeration on the ISET system. It is possible that the short time between blood-collections to blood processing may be critical to maintain CTC viability. The “bed-side” model of blood collection and processing implemented with the ISET system could therefore represent an operational advantage. The identification of higher number of CTCs in the ISET *vs*. CellSearch systems could also reflect the distinct CTC enrichment methodologies of the two platforms: antigen agnostic on the ISET platform, and EpCAM-dependent CTC enrichment on CellSearch. Non-epithelial or mesenchymal CTCs in blood from NSCLC patients would be detected uniquely on the ISET platform, but escape the antigen-dependent enrichment method of the CellSearch system [26].

The capacity of the ISET approach to detect CTCs in 75% of NSCLC blood samples allows molecular characterization of the tumor cells in a large proportion of advanced NSCLC patients. Several ICC and FISH assays have been developed in CTCs isolated and characterized using ISET [19, 27]. We reasoned that ISET would therefore provide an optimal platform to pursue MET assessment in CTCs. We developed an assay for MET analysis in ISET-enriched CTCs that uses the same antibody and MET staining protocol as the tissue-based IHC, and used concordant scoring algorithms to compute the MET status in CTCs and tissue. Using this assay, we found a strong correlation between MET status in tumor tissue and MET status in CTCs. Discordant MET status was observed in only 5 patients, all of which showed MET-positive CTCs and MET negative tumor assessed on whole tissue section, suggesting that in these cases CTCs might have originated from sites other than sampled tissue. By reflecting the metastatic disease process, CTCs may be more informative of biomarker status than a tissue biopsy taken at a given time [27]. This hypothesis could have important implications for developing new personalized strategies.

The relationship between MET overexpression and the outcome of NSCLC patients remains controversial, and may depend on the primary antibody used for MET assessment, cutoff criteria, and selection of samples (e.g. biopsy *versus* surgery samples) [28, 29]. The ISET study confirmed the prognostic value of CTC enumeration, and found no significant prognostic association of high MET protein expression in either tumor tissue or CTCs by using clinically applicable anti-MET antibody and well-defined scoring system according to the Onartuzumab study criteria. It is possible that the sample size of our cohort population along with bias of selection (according to positivity for CTCs) was not enough to provide statistical power to detect any survival impact of MET expression. Interestingly, MET was suggested to contribute to the putative metastasis-initiator circulating cells in breast cancer (e.g. EPCAM+CD44+CD47+MET+ CTCs, but not the bulk EPCAM+ CTCs) [30]. These findings do not exclude the possibility that other immunophenotypically defined CTC populations may contribute to poor outcome of patients.

In conclusion, we showed that CTCs from NSCLC patients are successfully detected on the ISET platform, and that MET status in ISET-captured CTCs correlate strongly with MET status in tumor tissue. This study provides a proof-of-concept for the potential use of CTCs as a sensitive and specific diagnostic testing of MET protein expression in patients with advanced-stage NSCLC that might be eligible for targeted therapies and could be of use to monitor therapy resistance. We are now expanding the CTC analysis to other biomarkers with relevance for lung therapeutics.

## MATERIALS AND METHODS

### Patients and samples

For the analysis of CTCs on the CellSearch system, peripheral blood samples were obtained from 256 Stage IIIB/IV NSCLC patients enrolled in Phase II clinical trials NCT01519804 (squamous NSCLC), and NCT01496742 (non-squamous NSCLC). Patients’ characteristics are summarized in Table 1.

For the analysis of patient-matched CTCs (ISET platform) and tumors, blood and tumor tissue samples were obtained from 106 newly diagnosed patients with histologically confirmed NSCLC and advanced-stage disease; these patients were treated at the Pneumology Department, (Pasteur Hospital, Nice) between January 2008 to December 2013. After CTC assessment, we selected 80 patients positive for CTCs for further analysis (see below); 20 patients presented initially with metastatic disease, and 60 patients presented with early-stage, but developed metastasis during treatment or follow-up. The main clinical and histopathological parameters of the 80 patients included in the study are summarized in Table 2. The institutional review board and local ethics committee of the University of Nice Sophia Antipolis approved this study. Informed written consent was obtained from all patients. Peripheral blood samples were available before surgery for all patients.

**Table 2:** Clinical and pathological characteristics of 80 pasteur hospital patients with advanced-stage NSCLC included in this study

Patients demographics (*n* = 80)	*N* (%)
**Age (*years***)	
Median (range)	65 (41–86)
**Sex**	
Male	54 (68%)
Female	26 (32%)
**Tobacco Use History**	
Former or current smokers	67 (84%)
Never smoked	13 (16%)
**Histological cell type**	
Invasive adenocarcinoma	63 (79%)
Squamous cell carcinoma	9 (11%)
Large cell carcinoma	3 (4%)
Sarcomatoid carcinoma	2 (2%)
Adenosquamous carcinoma	3 (4%)
**Tumor site sampling**	
Primary	60 (75%)
Metastatic	20 (25%)
**Median tumor size (range) cm**	2.5 (1–16)
**pTNM stage**	
IIIA	33 (41%)
IIIB	14 (18%)
IV	33 (41%)
**Differentiation grade**	
Well	17 (21%)
Moderate	19 (11%)
Poor	54 (68%)

Abbreviations: TNM = tumor node metastasis.

### Immunohistochemistry on tumor tissue

Automated IHC staining was carried out according to the manufacturer's protocol on the BenchMark XT platform (Ventana Medical Systems Inc./Roche Tissue Diagnostics, Tucson, AZ). IHC evaluation was performed using CONFIRM anti-Total c-MET (SP44) rabbit monoclonal primary antibody (catalog#790-4430, Ventana) and the ultraView Universal DAB Detection Kit (catalog#760-500, Ventana). Primary antibody was incubated for 16 minutes at 37°C. The MET IHC scoring was based on a combined assessment of membranous and cytoplasmic expression in tumor cells, and was evaluated blindly by three pathologists (MI, VH, and PH), with an overall concordance of 96%, using a qualitative 4-level intensity scale (0–3). Tumor cells showing MET protein intensities of 2 and 3 were considered positive. Sample status was determined using the Onartuzumab scoring criteria in lung: positivity was defined as having ≥ 50% of tumor cells positive for membranous and/or cytoplasmic MET immunostaining [31]. In addition, sample status was also determined using a weighted score (H-score) that takes into account the percentage of cells at each staining intensity (0–3+), resulting in a final H-score ranging from 0 to 300 [32].

### CTC enumeration and MET staining on the CellSearch platform

7.5 ml blood samples for CTC analysis were collected in CellSave tubes and immediately shipped to Genentech at ambient temperature. The median blood sample transit was 50 hours (range 18–988 hours), and only samples received before 96 hours from collection were analyzed (91% of total samples). Upon receipt, samples were processed for CTCs enumeration on the CellSearch platform using the CELLSEARCH^®^ Circulating Tumor Cell Kit, as per manufacturer's instructions (CellSearch, Inc.). CTC were identified following the CellSearch guidelines, and CTC enumeration was expressed as the number of CTCs detected in 7.5 ml blood. For the evaluation of MET expression, Alexa 488-conjugated MET antibodies (15A5 clone, Genentech, Inc.) were added to the open 4^th^ antibody position of the CellSearch system for automated MET staining. Normal blood samples spiked with control cell-lines that express high (EBC-1), medium (HCC1954) and low levels of MET (HCC70) were used as scoring controls for the quantification of MET expression in patients’ samples (Supplementary Figure 1). The level of MET expression was recorded for each CTC on a 0–3 scale, by comparison to the cell-line controls, with 3+ representing staining comparable to EBC1, 2+ comparable to HCC1954, and 1+ comparable to HCC70 cells. The sample's MET protein status was computed using a weighted H-scoring system, as above.

### CTCs enumeration and MET protein staining on ISET filters

CTC enrichment by ISET (Rarecells, Paris, France) was performed as per manufacturer's instructions. Briefly, 10 mL of blood were processed though the ISET filter within one hour of blood collection. The ISET filter contains 10 filter-spots, each representing the filtration of 1 ml blood. May-Grunwald-Giemsa (MGG) staining was performed on 4 filter spots, and these filter spots were examined for the presence of circulating non-hematological cells with malignant (CNHC-MF), uncertain (CNHC-UMF) and benign features (CNHC-BF), as previously reported [24, 33]. Samples that presented ≥ 1 CNHC-MF and/or CNHC-UMF in 4 ml sample were further tested for MET expression by immunocytochemistry (ICC) on 3 of the remaining unstained filter-spots [7], as follows: after 2 minutes of rehydration with Reaction Buffer 10x (catalog#950-300, Ventana), filters were placed in the BenchMark autostainer (Ventana), and followed the MET staining protocol as for IHC. Colored filters were mounted using Ultramount aqueous permanent mounting medium (catalog#S1964, Dako, Glostrup, Denmark). The MET ICC analysis assessed the membranous and cytoplasmic expression of MET in CTCs identified by the same morphological criteria used for the MGG analysis: size of the nucleus, anisonucleosis, nuclear-cytoplasmic ratio, presence of clusters. We also tested for false positive MET staining in healthy donor blood and in cell-spiking experiments processed on ISET, which were used for the development of ISET-based MET ICC. No leukocytes expressing MET were ever detected, and only rarely endothelial cells expressed MET in these samples. However, these cells are easily excluded by the rigorous cytopathology analysis that is part for CTC identification process. MET expression on ISET-enriched CTCs was scored on an intensity scale of 0–3 by comparison with control cell lines representing a range from high to low MET expression (H1993, A549, HEL293, MCF7, Supplementary Figure 2). MET protein status was determined using the Onartuzumab scoring criteria and a weighted H-score, as described for MET IHC. Results from CTCs and matched-tumor tissue were blinded until study completion.

### Statistical analysis

Concordance between tumor status and MET expression in CTCs was determined by Spearman rank correlation. Correlation coefficient ≥ 0.70 was considered good concordance. MET expression in tumor tissue and CTCs, analyzed as a binary positive vs. negative variable, was compared with clinico-pathological variables such as age, gender, smoking status, and tumor size, by using the χ^2^ analysis or the Mann-Whitney test when applicable. For outcome analysis, the primary end-point was progression-free survival (PFS) calculated from the time of histological diagnosis to the time of progression or death. Patients who were still alive at the end of study were censored at the end of study date. PFS was calculated using Kaplan-Meier method and a survival plot was generated.

## SUPPLEMENTARY MATERIALS FIGURES


