# Supplementary material for: Expression of MET in circulating tumor cells correlates with expression in tumor tissue from advanced-stage lung cancer patients
Source: Oncotarget. 2017 Feb 15;8(16):26112–21. doi: 10.18632/oncotarget.15345 (PMC5432243; doi:10.18632/oncotarget.15345)
Supplement: Supplementary file 1 [file oncotarget-08-26112-s001.pdf]

Expression of MET in circulating tumor cells correlates with expression in tumor tissue from advanced-stage lung cancer patients

Supplementary Materials

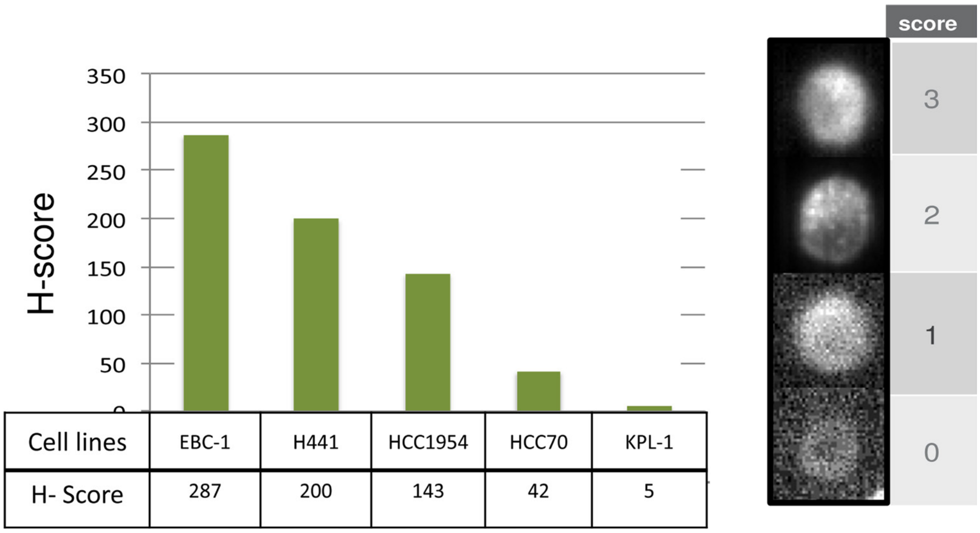

Cell lines	EBC-1	H441	HCC1954	HCC70	KPL-1
H- Score	287	200	143	42	5

Supplementary Figure 1: MET expression H-scores in cell lines EBC-1, H441, HCC1954, Hcc70 and KPL-1 spiked into healthy donor blood and processed through the CellSearch system. Right column: representative images of MET staining scored in a 0–3+ scale (right column). Bottom table: H-scores calculated for each cell line spike-in sample.

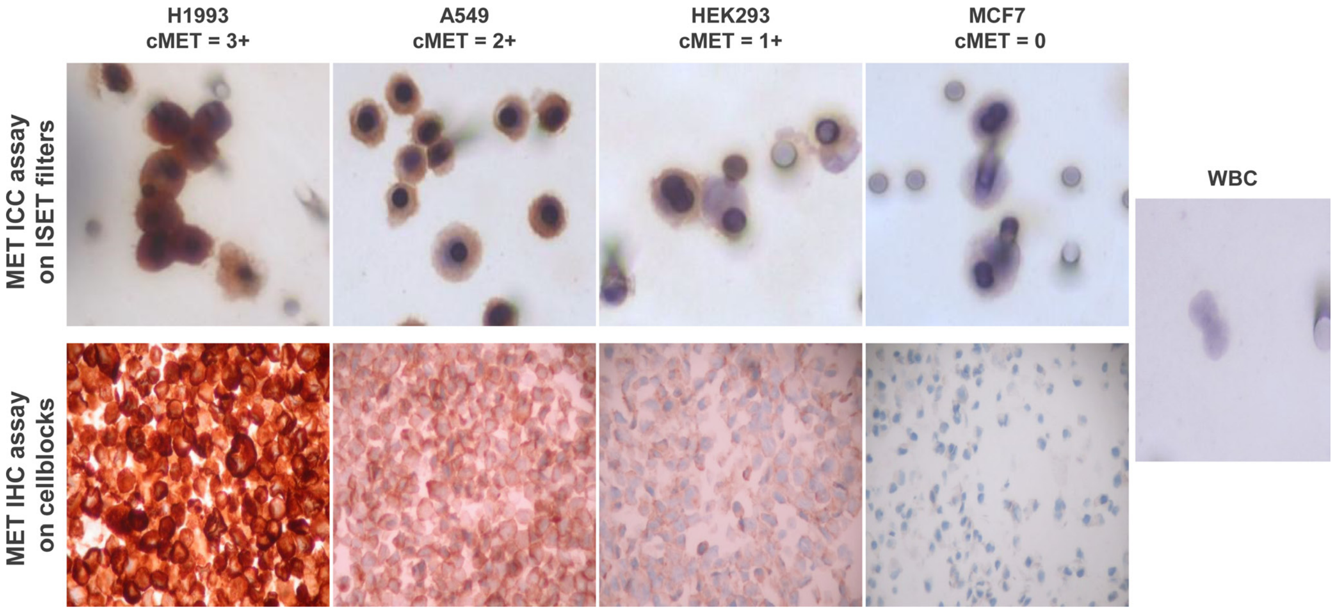

Supplementary Figure 2: Development of MET expression assay on CTCs. Cultured cells were spiked into healthy donor blood and isolated on ISET filters. MET expression was assessed on filters by immunocytochemistry (*upper panel*). Pellets from same cells were evaluated by IHC using the cMET SP44 assay (*lower panel*).

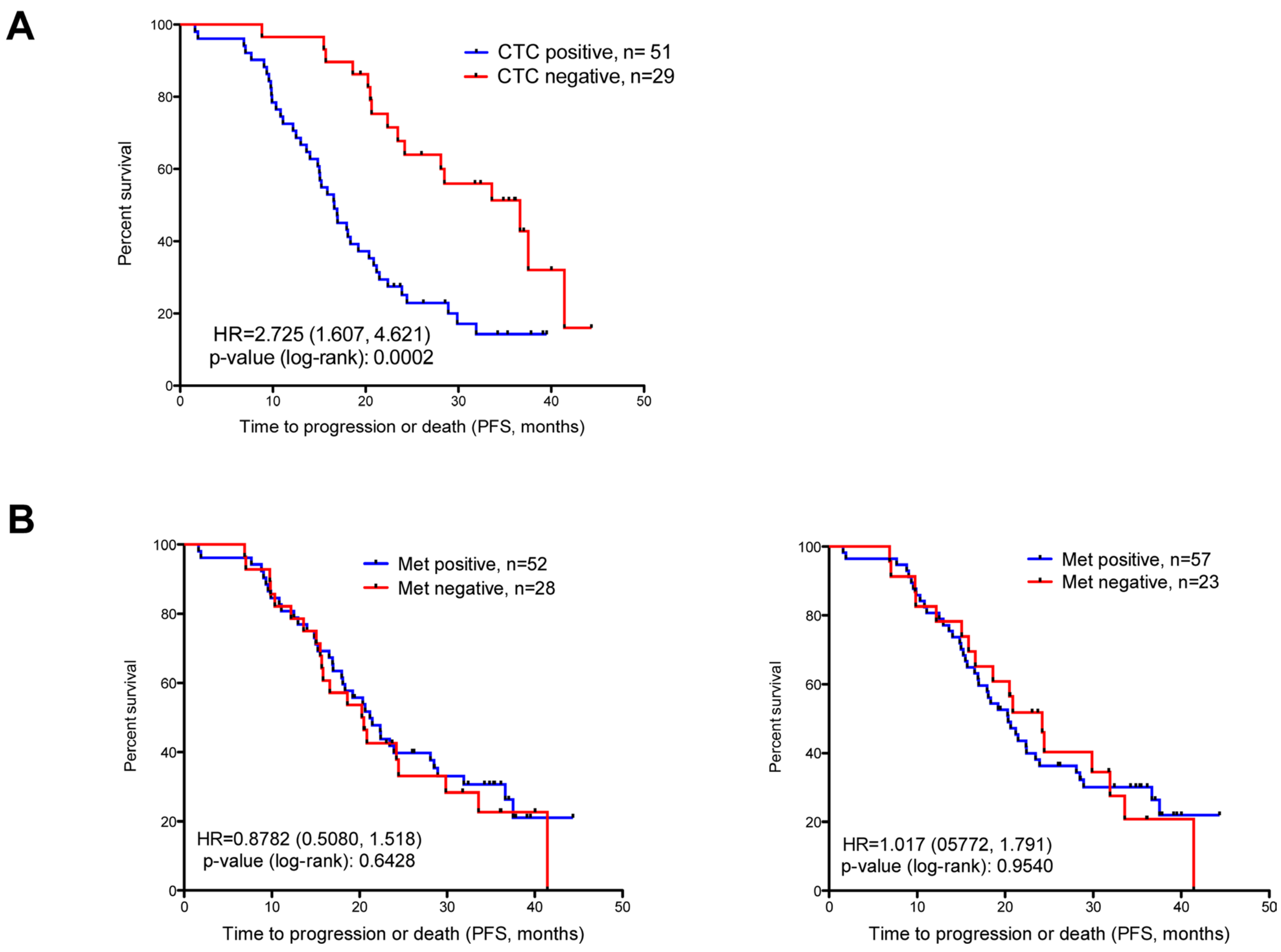

Supplementary Figure 3: Prognostic value of CTC enumeration and MET expression in patients from the Pasteur study. (**A**) Correlation between CTC enumeration by ISET and progression-free survival (PFS). (**B**) Correlation between MET expression in tumor tissue or CTCs isolated by ISET and progression-free survival (PFS). *Left,* PFS curve according to MET expression in tumor tissue; *Right,* PFS curve according to MET expression in CTCs.
